# Non-Invasive Genotyping of Honey Bee Queens *Apis mellifera* L.: Transition of the DraI mtDNA COI-COII Test to In Silico

**DOI:** 10.3390/insects12010019

**Published:** 2020-12-30

**Authors:** Shayne Madella, Kyle Grubbs, Mohamed Alburaki

**Affiliations:** USDA-ARS Bee Research Laboratory, Beltsville, MD 20705, USA; shayne.madella@usda.gov (S.M.); kyle.grubbs@usda.gov (K.G.)

**Keywords:** honey bee queen, genotyping, subspecies, Chelex DNA extraction, haplotype

## Abstract

**Simple Summary:**

Each honey bee colony has a single queen which plays a crucial role in the survival and wellbeing of the entire hive. Honey bee genetic analysis and selection and breeding programs rely on destructive methods using worker bees; workers are numerous in a colony and can be quickly replaced. In this study, we tested and validated a fast and efficient non-destructive method to conduct genetic analysis directly on honey bee queens. We also describe a new method for the transition to in silico of a widely used honey bee genetic marker by reconciling both cleaved amplified polymorphic sequences and Sanger sequencing approaches. Both new approaches will provide significant service to honey bee breeding and selection programs, as well as facilitating and standardizing honey bee haplotype identification among research institutions.

**Abstract:**

The honey bee *Apis mellifera* L. colony is headed by a single and indispensable queen, whose duty it is to ensure brood production and provide pheromonal stability within the colony. This study presents a non-invasive method that allows the identification of the queen maternal lineage and subspecies using the remaining tissue of her clipped wing. The DraI mtDNA COI-COII (DmCC) test was applied to various sizes of queen and worker wings and the results were compared with data obtained from other bee tissues. Furthermore, we propose a new method allowing in silico transition of the DmCC test and haplotype identification based on extended sequencing of the tRNAleu and COII genes. Our results show that DNA extracted by Chelex 10% from one-third of a queen’s wing is deemed adequate for a successful identification of her maternal evolutionary lineage, haplotype and subspecies. The in silico method proposed in this study fully adheres to the established guidelines of the DmCC, provides a universal standard for haplotype identification, and offers faster and more precise results by reconciling both cleaved amplified polymorphic sequences (CAPS) and Sanger sequencing approaches.

## 1. Introduction

The castes of a honey bee *Apis mellifera* colony comprise three different members; queen, workers and drones [1]. Under normal circumstances, the queen bee is the exclusive reproductive member in the colony and originator of all progeny. She is single and indispensable in her duty, providing pheromonal stability within the colony and ensuring the latter’s survival and growth. The poor quality of a queen, or queenlessness, induces significant disruption in the pheromone communication inside the colony [2] and could be detrimental if no requeening process is initiated promptly. Therefore, attempts to genetically characterize a given honey bee colony are always conducted on the queen’s offspring and the results are associated back to her by virtue. There are, however, some experimental circumstances in which identifying the genetic background of a queen by using her tissue is necessary, without dependence on her progeny or awaiting her egg-laying process to start. The availability of non-lethal sampling from queen bees would be valuable in many behavioral and patriline studies as well. Moreover, conducting genetic analysis directly on the queens’ DNA materials eliminates any potential ambiguity or doubt in the maternal origin of DNA extracted from offspring. Such a method must not produce any physical harm to the queen, which could severely impair her functions, leading to her supersession by worker bees. Clipping mated queen wings prior to introduction is commonly practiced by beekeepers and queen breeders [3]. This method is harmless for queens and offers an excised wing tip from the queen, which we used in experiments in this study.

The classification of honey bees and identification of subspecies relied initially on morphological characteristics such as wings’ cubital index and the number of hocks, colors, pilosity, tomentum and length of the proboscis [4,5,6,7,8]. Based on morphological characteristics, four honey bee evolutionary lineages were distinguished; West Mediterranean lineage M, North Mediterranean lineage C, African lineage A and Oriental lineage O [4]. These lineages were further confirmed by molecular markers, precisely mitochondrial and microsatellite markers [9,10,11,12,13,14,15,16,17,18]. Amongst these markers, the DraI mtDNA COI-COII (DmCC) test [13] stands as one of the most comprehensive mitochondrial markers widely used in honey bee genetic studies [19,20,21,22,23,24]. The principle of the DmCC test is conducting a PCR–RFLP, more precisely called the CAPS technique (cleaved amplified polymorphic sequence), on DNA extracted from honey bee tissues, in which the DraI enzyme is used for fragment restriction [10,11,13]. Since the initiation of this test, hundreds of honey bee haplotypes have been identified within all evolutionary lineages [16,19,20,25,26] and novel haplotypes are yet to be discovered in future studies. However, based on the principle of the DmCC test, to be qualified as a “novel haplotype”, alteration in patterns of the fragmented DNA must be visually identified on polyacrylamide gels. Eventually, SNPs or nucleotide substitutions within the restricted fragments would pass unnoticed by this test; a potential deficiency of the DmCC test. Thus, full sequencing of the mtDNA intergenic region COI-COII is usually the best remedy for this short coming. The DmCC test was designed as a quick method for the identification of honey bee evolutionary lineage, subspecies and haplotype without requiring a sequencing except in the case of novel haplotype confirmations. With the convenience of sequencing (low cost and rapid service), a transition of the DmCC test and previously identified haplotypes to in silico is necessary.

In this study, we tested a quick and straightforward method to extract DNA from the excised queen wing tips. The adequacy of the extracted DNA for genetic studies and sequencing was tested. Finally, we detail a new method that offers a full in silico transition of the DmCC test, cutting significant lab work while preserving the DmCC test’s rules for haplotype identification. This new method is based on reconciling both CAPS and sequencing techniques, which eliminates discrepancies in haplotype nomination and offers higher flexibility in haplotype comparison among research laboratories and studies.

## 2. Materials and Methods

### 2.1. Honey Bee Samples

**Figure 1:**
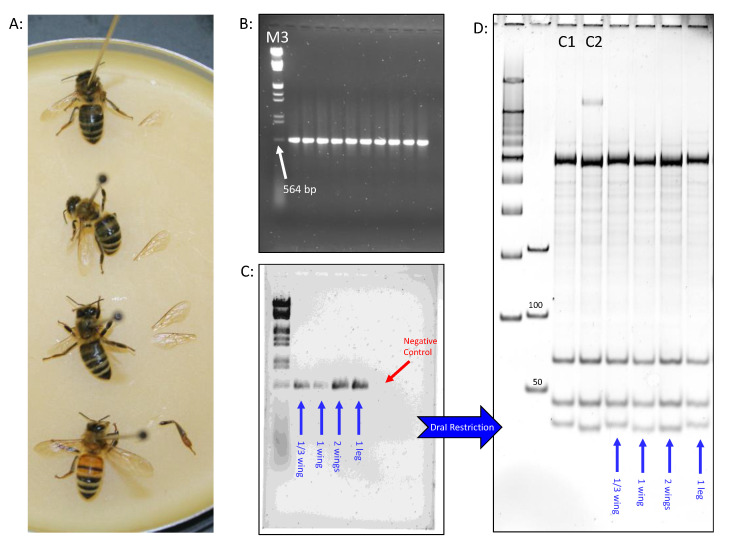
Outcome of the mtDNA genetic analysis conducted on various worker honey bees’ tissues. (**A**): a dissection of one-third, one and two wings, as well as one leg. (**B**): electrophoresis on 1.5% agarose gel of an optimal series of COI-COII PCR amplificons. (**C**): Same as (**B**) but for the studied tissues of (**A**). (**D**): Polymorphisms of the enzymatic restriction conducted on one-third, one, and two wings and one worker leg revealed on 7.5% polyacrylamide gel. The first four wells are two ladders and two reference haplotypes C1 and C2 which characterize *ligustica* and *carnica* subspecies, respectively.

All worker and queen bees used in this study were obtained from our current stock at the USDA-ARS Bee Research Laboratory at Beltsville, Maryland, U.S.A. Our apiaries are composed mainly of two honey bee subspecies; Italian *Apis mellifera ligustica* and Carniolan *Apis mellifera carnica*. Sixteen worker bees were randomly sampled from a few colonies and divided into four sets; each contained four biological replicates. DNA extraction was conducted on different sizes and types of tissues. We dissected one-third of a worker wing for the first set, one full wing for the second, two wings for the third and one leg for the fourth set (Figure 1). The same method was applied to 16 queen samples that were available and stored at −80 °C from a previous study. The thorax, head and two wings of an additional worker bee were dissected and prepared to be processed using a DNA extraction kit.

### 2.2. DNA Extraction

The primary method of DNA extraction used in this study on honey bee wings was the Chelex 10% [27] conducted in 96-well PCR plates. DNA extraction of honey bee samples was conducted as described in previous studies [19,20] but in 96-well PCR plates. In addition, and for comparison, DNA of three different tissue samples (wing, head and thorax) was extracted using a DNA extraction kit (GE Healthcare IllustraTM Triple Prep, Little Chalfont, UK) according to the manufacturer’s instructions. These tissues are frequently used in honey bee genetic studies. All extractions were subsequently nano-dropped for DNA quantity and quality.

### 2.3. Honey Bee Genotyping

Samples were genotyped by identifying their respective evolutionary lineage, haplotype and subspecies based on the widely used mtDNA COI-COII intergenic region marker and as described in many previous studies [9,10,11,12,13,26,28]. Briefly, the evolutionary lineage was revealed on 1.5% agarose gel using Lambda DNA/EcoR1 Molecular Marker (ThermoFisher, NY, USA) and the haplotypes with their respective subspecies were identified on 7.5% Polyacrylamide gels.

### 2.4. Haplotype Sequencing

In order to confirm the integrity of the DNA extractions conducted on queen bee wings, the haplotype polymorphisms and bee subspecies, the PCR amplicons of the mtDNA intergenic region COI-COII were sequenced using the traditional set of primer H2 and E2 [13]. Sanger sequencing was conducted by GENEWIZE company (South Plainfield, NJ, USA) and the alignment was carried out using GENEIOUS Prime software with reference sequences obtained from the National Center for Biotechnology Information (NCBI) Gene Bank of both C1 and C2 haplotypes (KX908209, HM117905).

### 2.5. Haplotype Profiling In Silico

The transfer of the haplotype patterns revealed on polyacrylamide gels to virtual gels requires the full sequence amplified by both H2 and E2 primers previously published [11,13]. To do so, we designed a new pair of oligos binding in both COI and COII genes beyond the H2–E2 amplified region from both ends. Sequences of the new oligo used in this study are COI_Seq-F: ACCACCTCTAGATCATTCACATTT and COII_Seq-R: AGGATGGAACTGTTCATGAATGAA. This new primer set (COI_Seq-F, COII_Seq-R) was used for the sole purpose of obtaining a full sequencing range of the H2–E2 region, thus allowing a transit toward in silico haplotype profiling by sequences according to the guidelines of the previously established DraI mtDNA test [11,12,13]. Note that this primer set is not intended and cannot be used to classically reveal DraI mtDNA haplotypes on polyacrylamide gels.

Four queen DNA samples were amplified using this primer and revealed on 1.5% agarose gel. PCR amplicons were sent to the GENEWIZE company (South Plainfield, NJ, USA) for sequencing. Sequences obtained were trimmed precisely at both H2–E2 edges and aligned using GENEIOUS Prime software (Geneious, CA, USA) along with the five reference samples characterizing different honey bee evolutionary lineages, haplotypes and subspecies (KX908209_ligustica, HM117905_carnica, Buckfast_C3, Haploytype_A2 and Haplotype_Z1). Using the same software, in silico DraI enzymatic restriction was applied on all these sequences and fragments were revealed on virtual gels with a ladder corresponding to the C2 haplotype patterns.

## 3. Results

### 3.1. DNA Extractions

In terms of concentrations, the DNA extracted from worker wings (Figure 1A) using the Chelex 10% method varied between 50–69 ng/µL (Table 1) and 40–139 ng/µL for the queen wings (Table 2). The extraction conducted on bee legs averaged 71 ng/µL for workers and 252 ng/µL for queen legs, (Table 1 and Table 2). The quantity of DNA extracted using the DNA extraction kit on bee wings was lower (20 ng/µL) than the average concentration obtained by Chelex (50–69 ng/µL) (Table 1). Although the DNA extractions carried out by the kit provided relatively better ratios, they remained far from optimal and generated lower DNA concentrations than what was obtained by Chelex 10%, as shown in Table 1.

### 3.2. CAPS Technique Integrity

**Figure 2:**
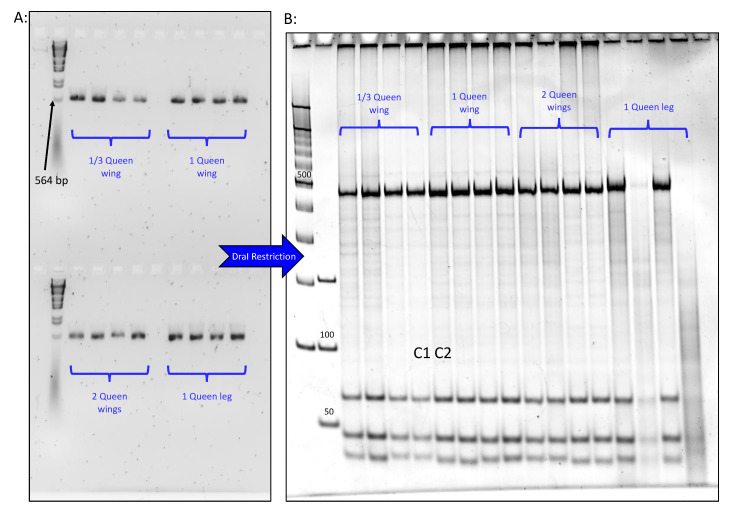
Results of the mtDNA genetic analysis conducted on queen wing and leg tissues. (**A**): PCR amplificons of the intergenic COI-COII region on 1.5% agarose gel, ladder is Lambda DNA/HindIII. (**B**): DraI restriction polymorphisms of the amplified intergenic region COI-COII revealed on 7.5% polyacrylamide gel. Queens exhibited both C1 and C2 haplotypes characterizing both honey bee subspecies; *ligustica* and *carnica*, respectively.

The cleaved amplified polymorphic sequence (CAPS) technique applied to all DNA extractions was intact, regardless of the tissues or samples used. Optimal results were obtained when conducting classic amplification of the intergenic region COI-COII on bee legs (Figure 1B), while slightly weaker signals were recorded for the worker wing tissues (Figure 1C). Despite relatively lower DNA quality from the worker wing extractions, enzymatic restriction with DraI revealed exemplary haplotype profiling (Figure 1D). Concerning the queen tissues, all extractions provided optimal mtDNA COI-COII amplifications, revealing a North Mediterranean lineage (C) for all queen samples (Figure 2A).

Regardless of the portion size, all queen and worker wings alike provided clear haplotype and subspecies identification on polyacrylamide gels with no unambiguity (Figure 2B). All samples exhibited either C1 and C2 haplotypes of the North Mediterranean lineage (C), which characterized both subspecies *Apis mellifera ligustica* and *A. m. carnica*, respectively.

### 3.3. Haplotype Sequencing

**Figure 3:**
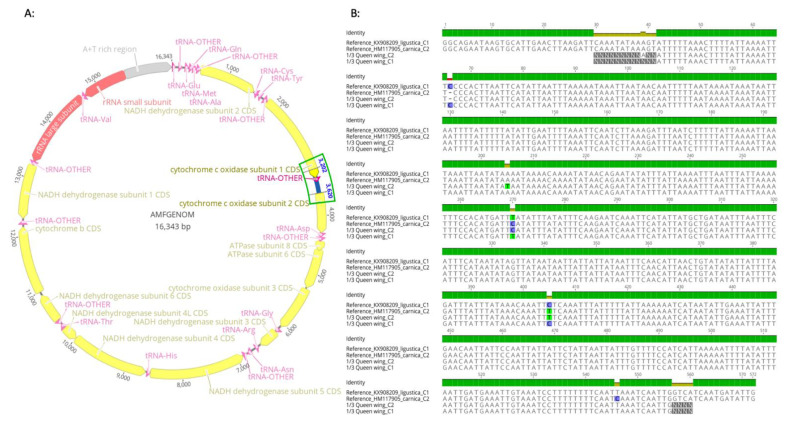
Structural organization of the honey bee mtDNA and COI-COII location. (**A**) Complete mitochondrial DNA genome of the honey bee *Apis mellifera* [29] annotated and showing the location of the intergenic region. (**B**) Successful sequencing of the mtDNA intergenic region using DNA extracted from one-third of a queen wing. Sequences were aligned with NCBI reference haplotypes C1 and C2 (KX908209, HM117905) using GENEIOUS Prime Software. Analyzed queens exhibited both C1 and C2 haplotypes, which correspond to *ligustica* and *carnica* subspecies, respectively.

The COI-COII intergenic region of the honey bee mtDNA [29] is detailed in Figure 3A. Two haplotypes identified on polyacrylamide gel as C1 and C2 and originated from one-third of a queen wing DNA extraction were sequenced using the traditional set of primer H2 and E2, Figure 3B. The sequencing using DNA extracted from wings was highly successful and showed no discrepancy or ambiguity in the nucleotide sequences, excluding potential alteration of poor DNA quality on the integrity of the sequencing (Figure 3B). Nonetheless, sequences of both H2 and E2 ends are not complete and cannot be obtained using this primer set as previously mentioned, Figure 3B.

### 3.4. Extended COI-COII Sequencing

**Figure 4:**
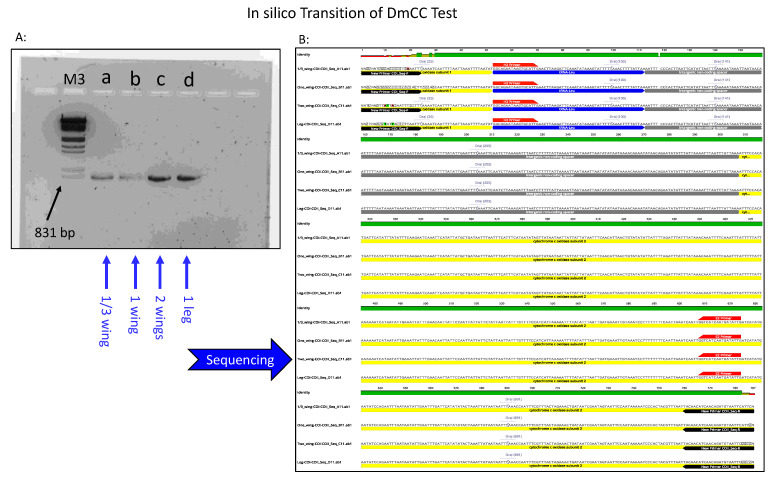
Transition of the DmCC test to in silico. (**A**): Amplification of the COI-COII region using a new proposed primer (COI_Seq-F and COII_Seq-R) revealed on 1.5% agarose gel. Ladder M3 is Lambda DNA/HindIII; samples a, b, c, and d, are one-third, one, two wings and one queen leg, respectively. (**B**): Sequences of the PCR amplicons showing the structural organization of the extended amplified region, location of the new primer (black), original primer (H2–E2) in red, cytochrome c oxidase subunit 1 and 2, tRNA-Leu, Q non-coding fragment and DraI restriction sites.

In order to abide by the guidelines of the DmCC test, we used the new primer set mentioned in Materials and Methods to obtain a full range of the COI-COII intergenic region. The new primer set (COI_Seq-F, COII_Seq-R) yielded amplicons of approximately 820 bp (Figure 4A) with sequences covering well beyond the two ends of H2 and E2 oligos. We annotated these sequences to show the structural organization of the mtDNA intergenic region COI-COII, which consists of tRNALeu followed by Q fragment and the Cytochrome Oxidase I gene as previously described (Figure 4B).

**Figure 5:**
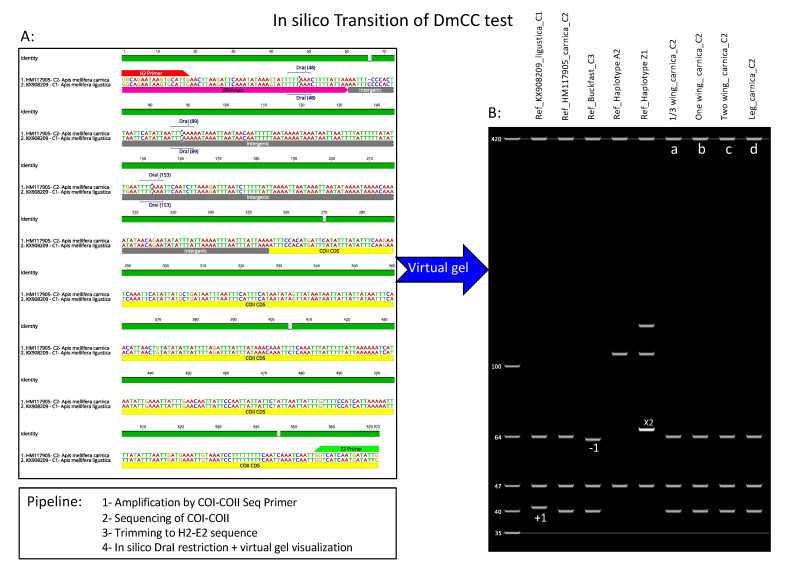
In silico procedures allowing the haplotype identification on a virtual gel. (**A**): trimmed sequence of the COI-COII intergenic at both H2–E2 ends and relevant DraI sites related to the DmCC test. (**B**): in silico DraI restriction and visualization of the fragment on a virtual gel. Reference haplotypes from the African lineage A (A2 and Z1) were added, as well as references of the North Mediterranean lineage C (C1, C2).

In order to reflect the haplotype profiling as required by the DmCC test and to allow possible comparisons with other studies using a universal standard, our extended sequences were trimmed precisely at both the E2 and H2 ends, as shown in Figure 5A. Using GENEIOUS software, DraI restriction sites were recognized in each sequence, Figure 5A. The final step of the DmCC test transition to in silico (Figure 5B) consisted of visualizing the restricted fragments on the virtual gel, as seen in Figure 5B. The four studied samples of the queen DNA extracted from wings (a, b, c, d) in Figure 4 are revealed on virtual gel along with five reference samples (Figure 5B). All the studied samples revealed the polymorphisms of C2 haplotypes, which indicates a Carniolan subspecies for these queens. The pipeline for this in silico transition is summarized by four steps: 1—PCR Amplification of the intergenic region by the new primer set; 2—Sequencing of the PCR products, 3—Trimming of sequences at H2–E2 ends; 4—In silico DraI restriction and virtual gel visualization.

## 4. Discussion

It should be mentioned first, that the aim of this study was not to compare different molecular markers used in honey bee genetic studies, nor to elaborate on the DraI mtDNA COI-COII (DmCC) test, because these topics have been extensively tackled in previous studies [13,30,31,32,33]. The DmCC test was previously conducted on DNA extracted from bee thorax and legs [19,20,28] using the Chelex 10% method [27]. The same extraction method and bee tissue was also used to successfully carry out various types of genomic analyses on honey bees [25,34,35]. Our results show that, whether for worker or queen bees, one-third of a wing is sufficient to conduct a comprehensive DmCC test leading to conclusive results, depicted in Figure 1 and Figure 2. It is evident that this technique might not be relevant for worker bees; they are numerous and can be destructively sampled. However, its relevancy is essential for queen bees because it harmlessly provides enough queen DNA for genetic analysis. In many situations, such as queen breeding and queen producer operations, the time required to obtain the queens’ offspring in order to precisely assess the genetic backgrounds and patrilines of mated-queens, as well as newly emerged queens (if artificial insemination is envisaged), may not be available. This method offers prompt genotyping, without relying on the queen’s descendants that could be misleading in some cases, such as sampling adult workers not related to the mother queen (robbing and drifting bees).

Wing clipping for mated queens prior to their introduction to colonies or packages is a common practice [3]. We demonstrated in this study that queen genotyping is possible using the remainder of the wing, leading to the determination of the evolutionary queen lineage, haplotype and subspecies. Despite not being the topic of this study, controversial arguments have been raised concerning the healthy practice of queen wing clipping. One would expect the wing DNA extraction to offer similar successful results for genomic DNA analysis, such as microsatellite or SNP analyses. A previous study indeed described a successful analysis of four microsatellite loci using DNA extracted from bee tip wings [36]. The current study, however, tested a much faster and straightforward method in plate DNA extraction (<3 h) than that proposed in the previous study [36].

The DmCC test was developed based on clear guidelines allowing the identification of various haplotypes according to visual fragment polymorphisms revealed exclusively on polyacrylamide gel [13]. As such, haplotypes were identified and named according to their DraI restricted fragments displayed on gels regardless of SNPs that did not alter the fragments’ sizes [13]. Rapid development and innovations in the biotechnology field, including high-throughput approaches to DNA, enable fast and cost-effective sequencing. Nonetheless, according to the conception of the DmCC test, designation of novel mtDNA haplotypes requires a visual display of new polymorphisms. It should not be based on a single nucleotide replacement, particularly as the region in question is a non-coding intergenic region. The failure to uphold this rule has created significant discrepancies in haplotype designation, which in turn has made data comparison among studies more challenging. Our method, proposed in this study, offers a dual advantage because it allows easy comparison and documentation of haplotype polymorphisms on virtual gels as well as in-depth analysis of any potentially occurring SNP in the haplotype sequences.

## 5. Conclusions

This study proposed and tested a fast and efficient method for non-invasive DNA extraction from queen honey bees, which allows for carrying out comprehensive mtDNA genetic analysis. We also detailed a new method for the transition to in silico of a widely used honey bee genetic marker by reconciling both cleaved amplified polymorphic sequences and Sanger sequencing approaches. This new approach will help to facilitate the transition of all previously identified haplotypes with their respective polymorphisms to virtual gels, which will allow more effective haplotype identification and comparison among studies.

## Figures and Tables

**Table 1:** Quantity and quality of the DNA extracted by the Chelex 10% method on honey bee worker wings and legs. Extractions were conducted on four biological replicates using a third of a wing, one full wing, two wings and a leg. UV Absorbance at 260 and 280 nm are presented as well as contamination ratios. DNA extracted from the wing, head and thorax using an extraction kit (GE Healthcare IllustraTM Triple Prep, Little Chalfont, UK) is provided for comparison.

Sample ID/Worker Bee DNA Extracted/Chelex 10%	ng/µL	A260	A280	260/280	260/230
One-third wing 1	36.21	0.724	0.68	1.06	0.22
One-third wing 2	38.20	0.764	0.59	1.30	0.20
One-third wing 3	88.71	1.774	1.12	1.59	0.43
One-third wing 4	39.75	0.795	0.54	1.47	0.29
Average	50.72	1.014	0.73	1.36	0.29
Full wing 1	32.47	0.649	0.58	1.11	0.21
Full wing 2	43.28	0.866	0.68	1.27	0.25
Full wing 3	38.50	0.77	0.67	1.15	0.23
Full wing 4	165.33	3.307	2.50	1.32	0.41
Average	69.90	1.398	1.11	1.21	0.28
Two wings 1	69.50	1.39	1.17	1.19	0.31
Two wings 2	43.21	0.864	0.69	1.25	0.23
Two wings 3	49.76	0.995	0.78	1.27	0.23
Two wings 4	85.52	1.71	1.09	1.57	0.36
Average	62.00	1.239	0.93	1.32	0.28
One leg 1	63.22	1.264	0.97	1.30	0.28
One leg 2	52.49	1.05	0.80	1.31	0.26
One leg 3	44.68	0.894	0.67	1.33	0.27
One leg 4	127.09	2.542	1.77	1.43	0.35
Average	71.87	1.437	1.06	1.34	0.29
DNA Extraction Kit					
Worker Wing	20.48	0.41	0.26	1.60	0.64
Worker Head	41.26	0.825	0.37	2.22	1.19
Worker Thorax	32.87	0.657	0.29	2.29	1.34

**Table 2:** Spectrophotometric results of Chelex 10% DNA extraction conducted on queen wings and legs. Tissues used were one-third of a queen wing, one full wing, two wings and one leg. Four biological replicates were tested and averaged for each category. The quantity of DNA is represented by (ng/µL), and DNA purity is reflected by both (260/280 and 260/230) ratios.

Sample ID/Queen Bee DNA Extracted/ Chelex 10%	ng/µL	A260	A280	260/280	260/230
One-third wing 1	66.86	1.34	1.06	1.27	0.26
One-third wing 2	184.16	3.68	2.52	1.46	0.38
One-third wing 3	39.25	0.79	0.62	1.28	0.27
One-third wing 4	38.19	0.76	0.60	1.27	0.25
Average	82.12	1.64	1.20	1.32	0.29
Full wing 1	35.46	0.71	0.58	1.22	0.25
Full wing 2	25.15	0.50	0.46	1.10	0.23
Full wing 3	60.25	1.21	1.01	1.20	0.27
Full wing 4	41.78	0.84	0.61	1.36	0.29
Average	40.66	0.81	0.67	1.22	0.26
Two wings 1	260.56	5.21	4.76	1.10	0.57
Two wings 2	191.53	3.83	3.68	1.04	0.40
Two wings 3	51.58	1.03	0.85	1.22	0.28
Two wings 4	54.29	1.09	0.87	1.25	0.26
Average	139.49	2.79	2.54	1.15	0.38
One leg 1	111.44	2.23	1.42	1.57	0.37
One leg 2	142.10	2.84	1.69	1.69	0.44
One leg 3	92.07	1.84	1.43	1.28	0.30
One leg 4	663.83	17.42	9.78	1.78	0.58
Average	252.36	6.08	3.58	1.58	0.42

## Data Availability

There is no additional data to disclose, all data are included in this manuscript.
